# Evaluating the Y chromosomal timescale in human demographic and lineage dating

**DOI:** 10.1186/2041-2223-5-12

**Published:** 2014-09-10

**Authors:** Chuan-Chao Wang, M Thomas P Gilbert, Li Jin, Hui Li

**Affiliations:** 1 State Key Laboratory of Genetic Engineering and MOE Key Laboratory of Contemporary Anthropology, School of Life Sciences, Fudan University, Shanghai 200438, China; 2 Centre for GeoGenetics, Natural History Museum of Denmark, University of Copenhagen, Copenhagen 1350, Denmark; 3 Department of Computational Genetics, CAS-MPG Partner Institute for Computational Biology, Shanghai 200031, China

**Keywords:** Y chromosome, Substitution rate, Demographic history, Lineage dating

## Abstract

Y chromosome is a superb tool for inferring human evolution and recent demographic history from a paternal perspective. However, Y chromosomal substitution rates obtained using different modes of calibration vary considerably, and have produced disparate reconstructions of human history. Here, we discuss how substitution rate and date estimates are affected by the choice of different calibration points. We argue that most Y chromosomal substitution rates calculated to date have shortcomings, including a reliance on the ambiguous human-chimpanzee divergence time, insufficient sampling of deep-rooting pedigrees, and using inappropriate founding migrations, although the rates obtained from a single pedigree or calibrated with the peopling of the Americas seem plausible. We highlight the need for using more deep-rooting pedigrees and ancient genomes with reliable dates to improve the rate estimation.

## Introduction

The paternally inherited Y chromosome has been widely applied in anthropology and population genetics to better describe the demographic history of human populations [1]. In particular, Y chromosomal single nucleotide polymorphisms (SNP) have been demonstrated one of the useful markers, thus have been widely used in genetic diversity studies over the last two decades [1]. One of the most important links between genetic diversity and human history is time, for instance, the time when a lineage originated or expanded, or when a population split from another and migrated. In this regard, molecular clock theory has provided an approach to build bridges between genetics and history. Specifically, under the assumption of substitution rate among lineages is constant, Y chromosomal molecular clocks have been used to estimate divergence times between lineages or populations [2-4]. Although this approach is widely accepted and used, there is still ongoing debate about the most suitable substitution rate for demographic and lineage dating [5]. In particular, there are several popularly used Y chromosomal substitution rates, such as the evolutionary rates measured from human-chimpanzee comparisons [6,7], the genealogical rate observed in a deep-rooting pedigree [8], the rate adjusted from autosomal mutation rates [9], and the rates based on archaeological evidence of founding migrations [10,11]. The choice of which kind of mutation rate to be used in Y chromosome dating is controversial, since different rates can result in temporal estimates that deviate several-fold. To address the above concern, we review how substitution rate and date estimates are affected by the choice of different calibration points.

## Review

### Y chromosome base-substitution rate measured from human-chimpanzee comparisons

In 2000, Thomson *et al.* screened three Y chromosome genes (*SMCY*, *DFFRY*, and *DBY*) for sequence variation in a worldwide sample set, using denaturing high-performance liquid chromatography (DHPLC) [6]. In order to infer the ages of major events in the phylogenetic trees, they had to first estimate the Y chromosome base-substitution rate. This they obtained by dividing the number of substitutions differences between a chimpanzee and human sequence over the relevant regions, by twice an estimated human-chimpanzee split time (5 million years) resulting in a substitution rate of 1.24 × 10^-9^ per site per year (95% confidence interval (CI) was not given in [6]). Using this rate, they were subsequently able to calculate the time of the Y chromosomal spread out of Africa to approximately 50 thousand years ago (kya) [6]. A weakness of this approach was that the sum of the lengths of the three genes was relatively small - at 64,120 base pairs (bp) it represented just a fraction of the total Y chromosome. Kuroki *et al.* attempted to address this in 2006, by sequencing nearly 13 Mb (more than 20% of the whole chromosome) of the male-specific region of the chimpanzee Y chromosome. Their analysis yielded a slightly higher rate, at 1.5 × 10^-9^ (assuming that the generation time is 30 years, 95% CI: 7.67 × 10^-10^-2.10 × 10^-9^), despite also using a chimpanzee-human calibration time that was 20% older than the previous study (6 million years) [7].

What is hopefully clear from the above, is that although direct comparisons of human and chimpanzee Y chromosomes offer us a powerful means to better understand the evolutionary process in our sex chromosomes during the past 5 to 6 million years, the process is clearly susceptible to a number of assumptions that need to be made. First, there is uncertainty over the exact timing of the human-chimpanzee divergence, as fossil records and genetic evidence have given a range of 4.2 to 12.5 million years ago [12]. Second, extreme structural divergence between the human Y chromosome and that of the chimpanzee makes it difficult to do precise alignment. The possible ascertainment bias and reference bias in data analysis might affect rate estimation. Third, it is not even clear that the human and chimpanzee Y chromosomes are even evolving under the same selective pressures. Specifically, the chimpanzee Y chromosome might be subject to more powerful selection driven by fierce sperm competition since the split of human and chimpanzee [13], which will accelerate the mutation rate in the chimpanzee lineage. Therefore, some concerns have been raised over whether the evolutionary rate based on human-chimpanzee divergence is consistent with the rate measured within human species or whether it can be used in human population demographic and paternal lineage dating.

Given the above, a variety of other methods have been proposed, including Y chromosome base-substitution rate measured in a deep-rooting pedigree, adjusted from autosomal mutation rates, and based on archaeological evidence of founding migrations. We address each of these in turn.

### Y chromosome base-substitution rate measured in a deep-rooting pedigree

In 2009, Xue *et al. *[8] sequenced Y chromosomes of two individuals separated by 13 generations using second generation paired-end sequencing methodology. Their analyses identified four mutations that had occurred over the 10.15 Mb of male-specific Y chromosome regions studied, enabling a base-substitution rate to be estimated as 1.0 × 10^-9^ per site per year (95% CI: 3.0 × 10^-10^-2.5 × 10^-9^) under the assumption that the generation time is 30 years. It is notable that this pedigree-based estimate overlaps with the evolutionary rates estimated from human and chimpanzee comparisons. For pedigree-based substitution rate estimation, there are at least two criteria to be taken into careful consideration. First, the pedigree must be biologically true and the generation information validated. The pedigree used by Xue *et al.* is a Chinese family carrying the *DFNY1* Y-linked hearing-impairment mutation. The same Y-linked disease-related mutation has validated the authenticity of their genealogy. Second, the detected mutations must be true. In this regard, Xue *et al.* used a variety of methods to verify the candidate mutations, thus validity of the rate: The Y chromosomes of the two individuals were sequenced to an average depth of 11× or 20×, respectively, thus mitigating the possibility of sequencing and assembling errors; they also reexamined the candidate mutations using capillary sequencing.

This pedigree-based rate has been widely used in Y chromosome demographic and lineage dating. Cruciani *et al. *[2] applied this rate to get an estimate of 142 kya to the coalescence time of the Y chromosomal tree (including haplogroup A0). Wei *et al. *[3] also used this substitution rate to estimate the time to the most recent common ancestor (TMRCA) of human Y chromosomes (haplogroups A1b1b2b-M219 to R) as 101 to 115 kya, and dated the lineages found outside Africa to 57 to 74 kya. Rootsi *et al. *[4] used this rate to estimate the age of R1a-M582 as 1.2 to 4 kya, suggesting the Near Eastern rather than Eastern European origin of Ashkenazi Levites.

Although this pedigree-based substitution rate is widely accepted, some concerns have also been raised. First, the mutation process of Y chromosome is highly stochastic, and the rate based on a single pedigree and only four mutations might not be suitable for all the situations. For instance, the haplogroup of the pedigree used in rate estimation of Xue *et al.* is O3a; however, other haplogroups probably have experienced very different demographic history and selection process, and might have different substitution rates as compared with haplogroup O3a. Second, the substitution rate was estimated using two individuals separated only 13 generations, thus, the question is whether the substitution rate estimated at relatively short time spans could be used in long-term human population demographic analysis without considering natural selection and genetic drift. Actually, many studies have noted that molecular rates observed on genealogical timescales are greater than those measured in long-term evolution scales [14].

### Y chromosome substitution rate adjusted from autosomal mutation rates

In 2013, in collaboration with the FamilyTreeDNA Company, Mendez *et al. *[9] identified a novel Y chromosome haplotype from an African American individual and the Mbo population living in Cameroon. This novel haplotype represents an out-group lineage to all other known Y haplotypes presently identified in human population. To estimate the time of origin of the novel haplotype, these authors neither used the existing rates for Y chromosome substitutions as estimated from human and chimpanzee comparisons [6,7] or from human deep-rooting pedigrees [8]; instead they developed a likelihood-based method that uses paternal autosomal mutation rates reported from an Icelandic data set of 78 parent-offspring trios. Under the assumptions that mutation rates are equal to substitution rates, and the Y chromosomal substitution rate is linearly related to the autosomal rate, they obtained a Y chromosome estimate of 6.17 × 10^-10^ per site per year (assuming that the generation time is from 20 to 40 years, range: 4.39 × 10^-10^ - 7.07 × 10^-10^). Strikingly, this substitution rate is only approximately half of the previous evolutionary rates and pedigree rate, although is very similar to estimates of autosomal rate [15]. In particular, it is unreasonable for the great disparity between Xue *et al.*’s pedigree rate and Mendez *et al.*’s rate which was also obtained from pedigree analysis. Mendez *et al. *[9] used his rate to argue for an extremely ancient TMRCA of human Y chromosomes as 338 kya (95% CI: 237to 581 kya), something inconsistent with the earliest fossils of anatomically modern humans (190 to 200 kya) [16]. While Mendez *et al. *[9] explained this discrepancy to long-standing population structure among modern human populations or archaic introgression from unknown species into the ancestors of modern humans in western Central Africa, other researchers have pointed out that the extremely ancient TMRCA could simply be attributed to the low substitution rate used by the authors [5]. Several reasons suggest that the Y chromosome mutation rate is expected to be higher than that of the autosomes. First it undergoes more rounds of replication in the male germline compared with autosomes [13]. In addition, long-term Y chromosomal substitution rates are not equal to single generation autosomal mutation rates, and purifying or advantageous selective pressures and genetic drift make it difficult to infer the correct Y chromosomal substitution rate from autosomal substitution rates [5]. Using the pedigree-based substitution rate results in a more reasonable estimate of TMRCA at about 208 to 209 kya [5-9], which is consistent with the earliest emergence of anatomically modern humans, and excludes the possibility of archaic introgression.

Elhaik *et al. *[5] also criticized the use of unreasonable generation times of Mendez *et al. *[9]*.* Mendez *et al. *[9] assumed that modern human had a paternal generation time ranging from 20 to 40 years, the upper band of which is even larger than the mean life expectancy of Cameroon men. The generation time is actually a key parameter in paternal lineage dating, as male mutation rates have been shown to increase with increasing generation time [5]. Rather than the range of approximately 20 to 40 years, Fenner has proposed a male generation length of 31 to 32 years through cross-cultural estimation [17]. The unreasonable generation times of Mendez *et al. *[9] seem to inflate the TMRCA estimate.

### Y chromosome base-substitution rate based on archaeological evidence of founding migrations

In 2013, Poznik *et al. *[10] reported the whole Y chromosome and mitochondrial genome sequences of 69 men from nine world-wide populations. Instead of using previous evolutionary and pedigree-based substitution rates for Y chromosome dating, they estimated the rate using a within-human calibration point, the initial migration into and expansion throughout the Americas. Well-dated archaeological sites indicate that humans first colonized the Americas about 15 kya [18]. A key assumption in this study was that the Native Amerindian Y chromosome haplogroups Q-M3 and Q-L54*(xM3) diverged at about the same time as the initial peopling of Americas. Using this, the authors obtained a mutation rate of 0.82 × 10^-9^ per site per year (95% CI: 0.72 × 10^-9^ to 0.92 × 10^-9^), and estimated the TMRCA of Y chromosomes to be 120–156 kya (haplogroup A1b1-L419). In comparison, the mitochondrial genome TMRCA was 99 to 148 kya. Thus the authors concluded that the coalescence times of Y chromosomes and mitochondrial genomes are not significantly different, which disagrees with the conventional suggestion the common ancestor of male lineages lived considerably more recently than that of female lineages [10]. The estimated Y-chromosomal substitution rate was subsequently applied to lineage dating within haplogroup R. The distribution of R1a and R1b, two main sublineages of haplogroup R, is suggested to be associated with recent episodes of population growth and movement in Europe. The divergence time of haplogroup R1a and R1b is estimated as 25 kya (95% CI: 21.3 to 29 kya) and a coalescence time within R1a-M417 is about 5.8 kya (95% CI: 4.8 to 6.8 kya) [19]. Similar to Poznik *et al.*’s calibration method, Francalacci *et al. *[11] also used archaeological records as a calibration point in lineage dating. Francalacci *et al. *[11] generated a high-resolution analysis of European Y chromosomes from population sequencing of 1,204 Sardinian men. They used the initial expansion of the Sardinian population about 7.7 kya as calibration point and the variation of all Sardinian individuals belonging to a subclade of haplogroup I2a1a to calculate a Y chromosomal substitution rate as 0.53 × 10^-9^ per site per year (95% CI: 0.42 × 10^-9^ to 0.70 × 10^-9^). This rate is extremely low and only half of the pedigree-based rate.

The main concern of the above two rates is the calibration point. In Poznik *et al.*, how do they know the Q-M3 and Q-L54*(×M3) diverged at the exact same time of initial peopling of Americas? In fact, individuals belonging to haplogroup Q-M3 have also been found in Siberia [20], suggesting the divergent event between Q-M3 and Q-L54*(×M3) probably occurred before the first colonization of Americas. An ancient genome of male infant about 12.6 kya recovered from the Anzick burial site in western Montana has helped to solve this dispute [21]. Y chromosome of this Anzick baby also belongs to haplogroup Q-L54*(xM3). By direct counting the transversions accumulated in the past 12.6 ky, Rasmussen *et al. *[21] estimated the divergence time of Q-M3 and Q-L54*(xM3) to be approximately 16.9 ky (95% CI: 13 to19.7). That is to say, the Y chromosomal substitution rate has been overestimated in Poznik *et al.* In Francalacci *et al.*’s case, the current Sardinian people might be directly descended from that initial expansion 7.7 kya, but there is also possibility that they are descended from a later successful founder population. If the latter is true, Francalacci* et al. *[11] have underestimated the substitution rate.

Although using the archaeological evidence for calibration in Y chromosomal substitution rate estimation is correct in principle, we have to pay great attention to whether the calibration point is reliable and suitable or not. In addition, more calibration dates could lead to more robust estimates. Besides the initial peopling of Americas and the initial expansion of the Sardinian population, the peopling of Oceania might be another good calibration point.

### Comparison of different Y chromosomal substitution rates in time estimation

**Figure 1:**
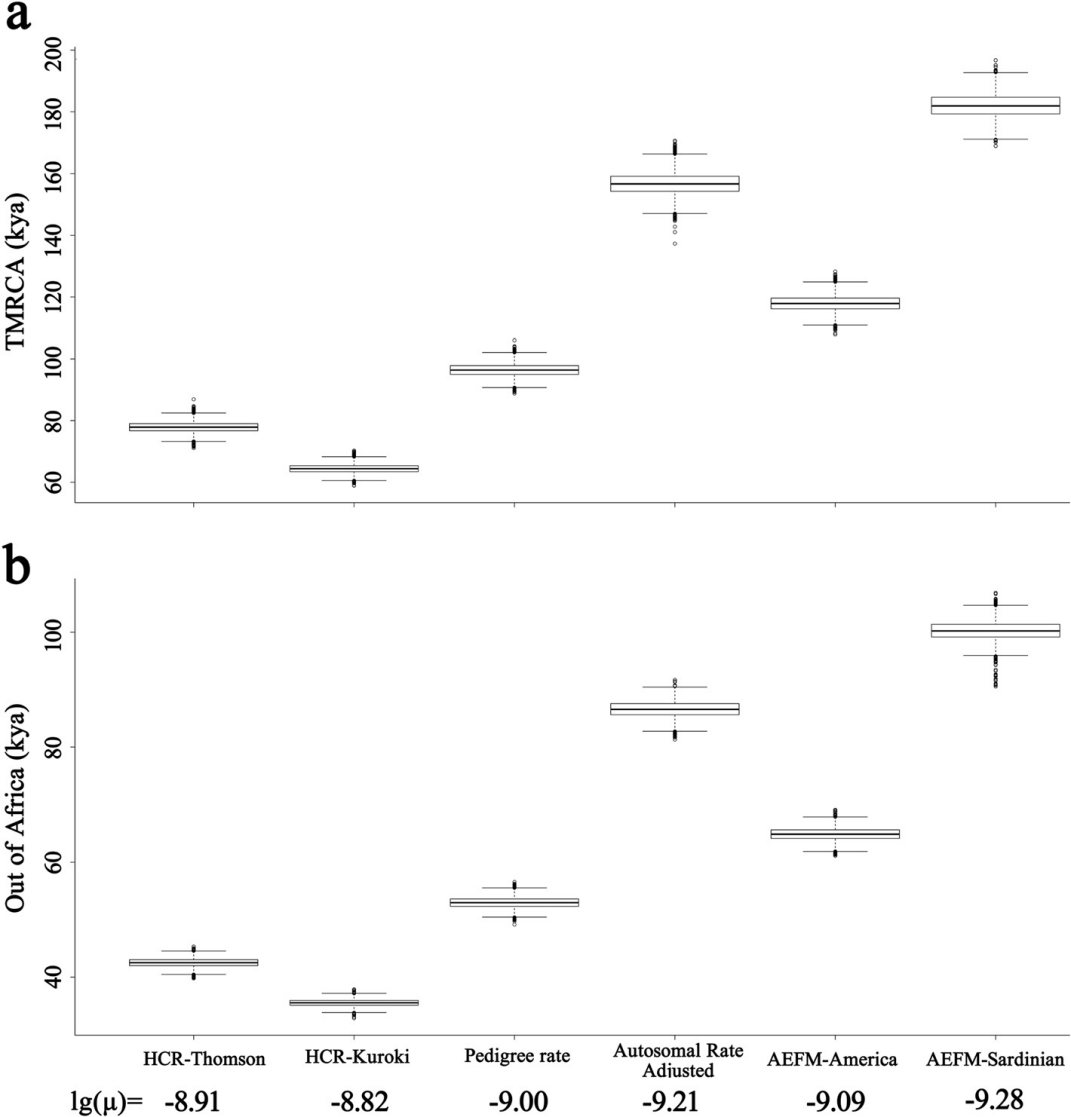
**Comparison of different Y chromosomal substitution rates in time estimation using Y chromosome dataset of 1000 Genome dataset.** Time estimations are performed in BEAST. **(a)** TMRCA of 526 Y chromosomes (including haplogroup A1b1b2b-M219 to T). **(b)** Time of Out-of-Africa migration, the age of macro-haplogroup CT. HCR- Thomson and HCR-Kuroki: Y chromosome base-substitution rate measured from human-chimpanzee comparison by Thomson *et al. *[6] and Kuroki *et al. *[7], respectively. Pedigree rate: Y chromosome base-substitution rate measured in a deep-rooting pedigree by Xue *et al. *[8]. Autosomal Rate Adjusted: Y chromosome substitution rate adjusted from autosomal mutation rates by Mendez *et al. *[9]. AEFM-America and AEFM-Sardinian: Y chromosome base-substitution rate based on archaeological evidence of founding migrations using initial peopling of Americas [10] and initial Sardinian expansion [11], respectively. Different reported mutation rates are given at the log scale. Confidence intervals for some of the mutation rates are very wide, and time calculations here use only the point estimate. The times would overlap more if all the uncertainties were taken into account. Figure was drawn using boxplot in R 3.0.2.

To simply illustrate the considerable effect of using the different proposed Y chromosomal substitution rates for me estimation, we used the Y chromosome dataset of 1000 Genome Project [22] to calculate both the Y-chromosome TMRCA, and the time of Out-of-Africa migration (Figure 1). The estimated TMRCA for the 526 total Y chromosomes (including haplogroup A1b1b2b-M219 to T) was 104.80 thousand years ago (95% CI: 100.20 to 109.58 kya) using pedigree rate, which is consistent with the published estimate of 105 kya [2] and 101 to 115 kya [3] for haplogroup A1b1b2b-M219 using pedigree rate. The next most important split point is the out-of-Africa superhaplogroup CT, which we date here at 52.96 kya (95% CI: 51.12 to 54.74 kya) using pedigree rate. However, the times estimated using rate based on archaeological evidence of initial Sardinian expansion is nearly two-fold of using pedigree rate, and almost three-fold of using rates obtained from human-chimpanzee comparisons. The times using rate calibrated by initial peopling of Americas are very similar with those applying pedigree rate, but still 10 to 20 ky larger. The rate adjusted from autosomal rates has inflated these time estimates by two-third as compared with pedigree rate. There are evidence for earliest modern human activities in Australia and neighboring New Guinea about 40 to 45 kya [23], in Southeast Asia about 37 to 38 kya [24], in China about 38 to 44 kya [25,26], and in Europe about 40 [27,28]. However, the time for Out-of-Africa migration estimated using rates obtained from human-chimpanzee comparisons are only 42.51 (95% CI: 40.96 to 43.98) and 35.50 (95% CI: 33.13 to 37.22) kya, which are smaller than the earliest archaeological evidence. Conversely, times estimated for Out-of-Africa migration using adjusted autosomal rate and rate calibrated by Sardinian expansion (86.56 and 100.22 kya, respectively) are 40 to 50 ky larger than the earliest modern human remains in the continents. Pedigree rate and rate calibrated by initial peopling of Americas produce more reasonable times for Out-of-Africa migration as 52.96 kya (95% CI: 51.12 to 54.74 kya) and 64.89 kya (95% CI: 62.64 to 67.12 kya). Those results are very consistent with our above assumptions. The rates measured from human-chimpanzee comparisons are probably slightly higher than real human Y chromosomal substitution rates as the fierce sperm competition has accelerated the mutation rate in the chimpanzee lineage. The adjusted autosomal rate is lower than real Y chromosomal substitution rate due to fewer rounds of replication in autosomes compared with male germline. Rate calibrated by Sardinian expansion might be also lower than the real rate probably due to the current Sardinian people are descended from a later successful founder population rather than from the initial expansion 7.7 kya. The pedigree rate and the rate calibrated by initial peopling of Americas might be slightly higher than real substitution rate, but it still need more evidence to prove.

## Conclusions

Some of the most widely-cited Y chromosomal substitution rate estimates have several shortcomings, including a reliance on the ambiguous human-chimpanzee divergence time, insufficient sampling of deep-rooting pedigrees, and using inappropriate founding migrations. Here, we propose two possible approaches to obtain greater precision in measuring Y chromosomal substitution rate. First is the pedigree-based analysis, we can collect and sequence some reliable deep-rooting pedigrees representing a broad spectrum of worldwide Y chromosomal lineages or at least common haplogroups of East Asia. Recording the family trees has been a religious tradition of Han Chinese, and some family trees even span more than 100 generations, linking the contemporary individuals to their ancestors over 2 to 3 kya, although their authenticity requires careful validation [29,30]. More reliable deep-rooting pedigrees could overcome the possible bias in rate estimation caused by previous one single pedigree and only four mutations as we have discussed above. An alternative approach is through the sequencing of Y chromosomes from ancient samples for which reliable radiocarbon dates are available, something previously demonstrated for calculating the human mitochondrial substitution rate by the Krause lab. They applied the mitochondrial genomes of 10 securely dated ancient modern humans spanning 40 ky as calibration points, thus yielding a direct estimate of the mitochondrial substitution rate [31]. With the fast emerging and growing ancient DNA analysis techniques, entirely sequenced Y chromosomes in ancient individuals have become available, for instance, the 24-ky-old Siberian individual with haplogroup R [32], the 12.6-ky-old Anzick infant of Q-L54* [21], the 7-ky-old Mesolithic European belonging to haplogroup C6 [33], the Mesolithic Swedish hunter-gathers with haplogroup I2a1 [34], and the 4-ky-old Palaeo-Eskimo with haplogroup Q1a-MEH2 [35]. The Y chromosome sequencing of ancient samples, although promising, still has to overcome many hurdles, such as low coverage, possible contamination or ascertainment problems. However, we remain optimistic that the ancient DNA approach will change this awkward situation for Y chromosomal substitution rate estimates.

## Abbreviations

bp: base pairs; CI: confidence interval; DHPLC: denaturing high-performance liquid chromatography; kya: thousand years ago; SNP: single nucleotide polymorphism; TMRCA: time to the most recent common ancestor.

## Competing interests

The authors declare that they have no competing interests.

## Authors’ contributions

CCW conceived of the study, performed data analysis and wrote the manuscript. HL supervised the study and assisted in drafting and revising the manuscript. MTPG, LJ are involved in the discussion and revised the manuscript. All authors read and approved the manuscript.
